# An integrative evaluation of drying methods in relation to bioactive properties and metabolomic profiles of four *Nymphaea* cultivar petals

**DOI:** 10.1016/j.fochx.2026.104468

**Published:** 2026-09-19

**Authors:** Xuhong Zhou, Jiangli Peng, Qianqian Li, Fujun Zheng, Min Tian, Suping Qu

**Affiliations:** a Research Experiment Center, Yunnan University of Chinese Medicine, Kunming 650500, China; b Xishuangbanna Baocui Xianglian Biotechnology Co., Ltd., Xishuangbanna 666212, China; c Flower Research Institute, Yunnan Academy of Agricultural Sciences, Kunming 650500, China

**Keywords:** *Nymphaea* spp., Drying methods, Edible flowers, Biological activities, 1,2,3,4,6-*O*-Pentagalloylglucose

## Abstract

*Nymphaea* species (water lilies) exploited for their dual use as food and medicine. We systematically quantified the quality and bioactive properties activities of four water lily petals subjected to different drying methods. Our findings showed freeze drying maintained the superior external quality and aroma of the petals, whereas shade drying was successful in preserving bioactive compounds like 1,2,3,4,6-*O*-pentagalloylglucose (PGG) in most cultivars. In contrast, microwave oven drying exhibited superior antioxidant, antibacterial, and tyrosinase inhibitory activities. As a potential key functional compound, PGG possesses multiple bioactive properties, including antioxidant, whitening, lipid-lowering, antidiabetic and anti-tumor effects. *Nymphaea* ‘Blue Bird’ is promising as a functional food candidate, attributed to its high accumulation of PGG. The metabolite profiles of *N*. ‘Blue Bird’ were analyzed using non-targeted metabolomics with full-MS/MS analysis. During drying, the DAMs were mainly linked to ABC transporters and tryptophan metabolism. Collectively, these findings provide a scientific basis for optimizing industrial processing protocols.

## Highlights

• MD is optimal and boosts bioactivities.
• FD preserves quality and fragrance.
• SD retains abundant bioactive compounds.
• PGG possesses multiple bioactive properties.
• *N*. ‘Blue Bird’ has great functional food potential.

## 1 Introduction

*Nymphaea* species (water lilies), a perennial aquatic flower plant, are commonly used in landscaping for decoration and are also traditionally consumed as food (Afrin et al., 2024; Wasagu et al., 2015). In the medical field, water lily stems, roots, and flowers, are utilized for treating tuberculosis, coughs, inflamed glands, and mouth sores to prevent bleeding (Wasagu et al., 2015), and are used as a therapy for diabetes (Prodhan & Mridu, 2023). The analysis of water lilies reveals that they contain numerous bioactive substances such as flavonoids, phenolics, tannins, anthocyanins, saponins, glycosides, triterpenes, and other compounds, which contribute to a variety of pharmacological effects (Abelti et al., 2023; Prasad & Savithramma, 2016). The antioxidant capacity of *N. lotus* petal ethanolic extracts was found to be similar to that of ascorbic acid, according to the DPPH assay (Semaming et al., 2018). Flowers from *N. pubescens* are reported to have antidiabetic properties (Pokhrel et al., 2022). The ethanolic extracts from *N. pubescens* flowers were found to be toxic to human breast cancer MCF and cervical cancer Hela cell lines (Selvakumari et al., 2012). According to Kamma et al. (2019), the ethanol extracts from the stamens and petals of *N. rubra* have been found to have skin lightening and anti-aging effects. The methanolic extracts from *N. mexicana* flowers showed potential as cholinesterase inhibitors (Sabreen & Masoodi, 2019). Chemometric analysis identified gallic acid, a known metabolite, as a significant contributor to the inhibition of AChE by *N. pubescens* flower extracts (Acharya et al., 2018). Additionally, *Nymphaea* species bloom repeatedly and emit a strong scent, with alcohols, alkenes, and alkanes as the main volatile compounds in their petals (Zhou et al., 2022). Thus, water lily flowers hold significant potential for development in the tea beverage industry due to their high ornamental value and health benefits.

Drying is a traditional preservation method that prevents microbial growth, enzymatic browning, and spoilage while maintaining active components and floral tea quality (Fan et al., 2025). Due to the rising demand for superior quality products, cost-efficient processes, and minimal environmental effects, drying technology has garnered significant attention and research (Mujumdar & Law, 2010). In the herb industry, shade drying (SD) and hot air drying (HD) are popular because they are simple and inexpensive, whereas vacuum freeze drying (FD) offers the low temperature and oxygen conditions needed to maintain herb quality. Microwave drying provides benefits such as improved drying speed, precise regulation, quick initiation and cessation, and high-quality dried products (Setiady et al., 2009). Research indicates that this drying method changes the chemical makeup and biological properties (Bian et al., 2024; Park et al., 2021). To maximize the yield of desired phytochemicals, it's important to choose the right drying method, as not all developed drying techniques are suitable for different types of biological materials (Chen et al., 2024; Park et al., 2021).

There is limited research on how different drying techniques for water lily flowers affect metabolite content, antioxidant properties, and enzyme inhibition activities. Unlike previous studies that focused on single cultivars or limited quality traits, our work provides an integrated metabolomic and physicochemical evaluation across four *Nymphaea* cultivars and four drying methods. We assessed changes in appearance, aroma, and key chemical components (polysaccharides, vitamin C, amino acids, phenolics, anthocyanins, and flavonoids) of water lilies during drying, and employed untargeted metabolomic strategies utilizing LC-MS/MS and GC–MS to profile compound transformations. Based on these analyses, this study not only provides the first comprehensive evaluation of drying effects on the chemical and bioactive profiles of *Nymphaea* petals, but also identifies PGG as a prominent bioactive marker warranting further investigation. These findings may facilitate the use of water lily in food, medicinal, and tea product applications.

## 2 Materials and methods

### 2.1 Plant materials

Four *Nymphaea* cultivars, namely ‘Eldorado’, ‘Blue Bird’, ‘Purpledo’ and ‘Ruby’, were cultivated in Xishuangbanna Dai Autonomous Prefecture, Yunnan Province, China. Fresh flowers were harvested in March 2024 (Fig. 1A–D). After harvesting, the fresh petals (FP) were processed using four different drying methods: indoor shade drying (SD), microwave oven drying (MD), hot air drying (HD) and freeze drying (FD). Each experimental group underwent three independent replicates, which were processed simultaneously. Fresh flowers were lyophilized in a SCIENTZ-18 N/C freeze-dryer (Ningbo Scientz Biotechnology Co., Ltd., China) at −50 °C for 48 h for the FD treatment. HD was performed in a GZX-9240MBE electric thermostatic drying oven (Shanghai Boxun Medical Biological Instrument Co., Ltd., China) at 60 °C for 4 h. Microwave treatment involved drying samples in a PM2002 microwave oven (Midea Group Co., Ltd) at 800 W for 2–3 min. Shade drying was performed in a well-ventilated area at room temperature over a 3 days period. All prepared samples were finely ground and stored at −20 °C until subsequent analysis.

**Fig. 1:**
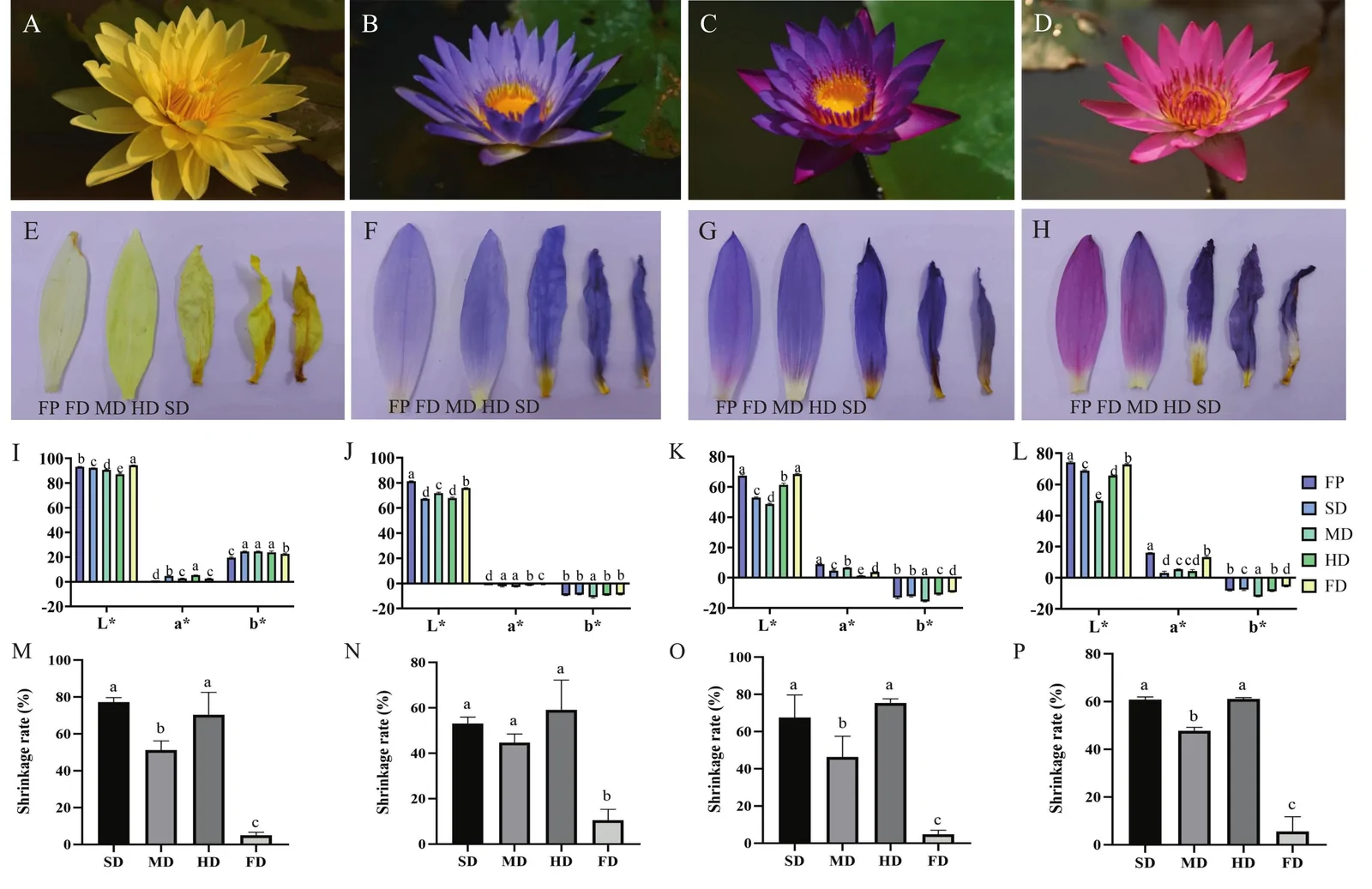
Phenotypic and quantitative analysis of water lily petals subjected to different drying treatments. A-D Morphology of fresh petals from four *Nymphaea* cultivars: (A) *N.* ‘Eldorado’; (B) *N*. ‘Blue Bird’; (C) *N*. ‘Purpledo’; (D) *N*. ‘Ruby’. *E*-H Visual comparison of petal appearance after four drying treatments. I-L Quantitative analysis of color parameters of dried petals. M–P Shrinkage rate of dried petals under different drying treatments. Each panel of E-H, I-L and M-P corresponds to the cultivar in A-D, respectively. Distinct lowercase letters denote significant differences between treatments (*P* < 0.05). Values are presented as the mean ± standard deviation (SD) from three replicates. Abbreviations: FP, fresh petal; SD, indoor shade drying; MD, microwave drying; HD, hot air drying; FD, freeze drying. (For interpretation of the references to color in this figure legend, the reader is referred to the web version of this article.)

### 2.2 Analysis of the aroma components of water lily petals

Five grams of fresh and dried water lily petals from the four varieties were prepared separately. The samples were transferred to a 100 mL conical flask and covered with sealing film for 30 min. The analysis of aroma components in water lily petals was measured using an electronic nose (e-nose), with some minor modifications based on previous descriptions (Sun et al., 2018). The PEN3.5 e-nose was pre-warmed for 15 min prior to each analysis session. The sampling run time sufficient for the sensors to reach a stable value was 100 s. After resetting the sensor baseline for a minute, the array began gathering readings every two seconds. The headspace gas sample was circulated across the sensors at a flow rate of 400 mL per minute, with the entire process managed by Winmuster software (AIRSENSE ANALYTICS GmbH, Schwerin, Germany). The specific flavor compounds detected by each of the e-nose's ten sensors are outlined in Table S1.

### 2.3 Evaluation of appearance

Appearance attributes including color indices and shrinkage ratio of the dried samples were determined. Flower color parameters (L*, a*, b*) were measured with a ST60 chroma meter (Shenzhen Threenh Technology Co., Ltd., China). Shrinkage ratio was quantified following the method reported by Chen et al. (2018).

### 2.4 Determination of standardized quality parameters

The total flavonoid content (TFC), the total phenolic content (TPC), and the total anthocyanin content (TAC) of water lily flower was calculated according to procedures described previously (Tao et al., 2025). The total phenolic content (TPC) was quantified as milligrams of gallic acid equivalents per gram of either dry or fresh weight of water lily (mg/g). Similarly, the total flavonoid content (TFC) was measured as milligrams of rutin equivalents per gram of dry or fresh weight (mg/g), and the total anthocyanin content (TAC) was determined as milligrams of cyanidin-3-*O*-glucoside equivalents per gram of dry or fresh weight (mg/g).

Precisely 0.5 g of both fresh and dried water lily petal samples were separately weighed. Subsequently, 50 mL of boiling water was added to each sample for a 30-min extraction period, followed by filtration. Reduced vitamin C (VC) was quantified via the molybdenum blue method, while free amino acids were determined by the ninhydrin method with a standard curve established using glutamic acid (Chai et al., 2023).

0.5 g of petal powder was mixed with 25 mL disodium hydrogen phosphate-citric acid buffer (pH 6.8), followed by the addition of 1% (w/w) complex enzyme (cellulase: papain = 1:1, w/w). The solid–liquid ratio was set at 1:50 (m/v), and extraction was conducted at 50 °C for 100 min. After rapid heating for enzyme inactivation, the extract was cooled and filtered. The total sugar content of petals was determined by the phenol‑sulfuric acid method (Song et al., 2022).

### 2.5 Evaluation of antioxidant capacity

Antioxidant activity was assessed using ABTS, DPPH, and FRAP assays as previously described by Tao et al. (2025).

### 2.6 Enzyme activity

The chromogenic DNSA method outlined by Sudha et al. (2011) was used to assess the α-amylase inhibitory activity. In the experiment, solution of each sample was diluted to different concentrations, including 4, 2, 1, 0.5, 0.25, 0.125 and 0.0625 mg dry weight or fresh weight /mL. The lipase assay followed the method of Mhatre et al. (2019) with some modifications, and it was conducted in triplicate on a 96-well microtiter plate. A solution of pancreatic lipase enzyme (10 mg/mL) from pig pancreas was prepared in water and kept at 2–8 °C until it was used. The substrate used in this test was *p*-nitrophenyl palmitate, initially dissolved in dimethyl sulfoxide for complete solubilization. Ultra-pure water was then added to bring the solution concentration to 10 mmol/L. The mixture, consisting of 20 μL of sample, 20 μL of pancreatic lipase, 140 μL of 0.05 M Tris-HCl buffer (pH 7.4), and 20 μL of *p*-nitrophenyl palmitate solution, was incubated at 37 °C for 20 min, and absorbance was recorded at 405 nm. The inhibition rate of pancreatic lipase was determined using formula (1).

(1) $$\text{Inhibition rate}\;\left(\%\right)=\left[A-\left(B-C\right)\right]/A\times 100\;$$

where blank group A indicates the absorbance of 20 μL enzyme and 20 μL substrate, 160 μL Tris-HCl, sample group B indicates the absorbance of 20 μL sample, 20 μL enzyme, 20 μL substrate and 140 μL Tris-HCl, sample control group C indicates the absorbance of 20 μL sample and 180 μL Tris-HCl.

Tyrosinase inhibitory activity was assessed. Briefly, a 50 μL sample was combined with 25 μL of tyrosinase solution and incubated at 37 °C for 10 min. Afterward, 100 μL of a 1 mol/L L-DOPA solution was added to start the reaction, and the absorbance was recorded at 475 nm. Kojic acid was used as the positive control. The rate of tyrosinase inhibition was determined using a formula (2).

(2) $$\text{Inhibition rate}\;\left(\%\right)=\left[1-\left(\mathrm{Ab}-A\right)/\left(\mathrm{Cb}-C\right)\right]\times 100$$

where Ab is the absorbance of the 50 μL sample solution with 25 μL tyrosinase solution, A is the absorbance of the 50 μL sample solution with 25 μL sodium phosphate buffer (pH 6.8), Cb is the absorbance of the 50 μL buffer (pH 6.8) with 25 μL tyrosinase solution, and C is the absorbance of the 75 μL buffer alone (pH 6.8). All reaction systems require the addition of 100 μL of a 1 mol/L L-DOPA solution as the substrate.

### 2.7 Determination of analytes

The four key components of *Nymphaea* species, including methyl gallate, gallic acid, geraniin and 1,2,3,4,6-*O*-pentagalloylglucose, were precisely quantified using HPLC. Each sample was analyzed for four analytes using high-performance liquid chromatography (HPLC) with an Agilent Series 1260 LC instrument from Agilent Technologies, USA. Chromatography was conducted using an Agilent ZORBAX SB-C18 column (250 mm × 4.6 mm, 5 μm). The HPLC methods were adapted from previously reported procedures (Zou et al., 2023) with minor modifications. The mobile phase was made up of solvent A (0.2% phosphoric acid in water, *v*/v) and solvent B (acetonitrile). The gradient elution schedule was: 5–15% A from 0 to 35 min, and 15–18% A from 35 to 65 min, 18–20% A from 65 to 70 min and 20–5% A from 70 to75 minutes. The flow rate was set at 1.0 mL/min with an injection volume of 10 μL. The column was kept at a temperature of 30 °C. The diode-array detector was adjusted to 266 nm for both methyl gallate and gallic acid, geraniin and 1,2,3,4,6-*O*-pentagalloylglucose, respectively. Standard stock solutions of the four analytes were created and subsequently diluted to suitable concentrations for calibration and method validation.

The dried powders (∼0.5 g) were sonicated in 80% methanol (50 mL) at 60 °C for 45 min. The combined supernatants were concentrated using rotary evaporation to enhance the active extract, then diluted with 80% methanol to reach a final volume of 25 mL. Before HPLC analysis, the samples were filtered through a 0.22 μm microfiltration membrane. Each analyte's concentration was measured three times and calculated based on the fresh or dry weight of the sample.

### 2.8 Determination of antimicrobial activity

The study assessed the antimicrobial properties of the *Nymphaea* species extract using the disk diffusion technique (Tao et al., 2025). Three different bacterial species (*Escherichia coli*, *Pseudomonas aeruginosa* and *Staphylococcus aureus*) were chosen for the antimicrobial activity test.

### 2.9 Determination of anticancer activity

1,2,3,4,6-*O*-pentagalloylglucose was evaluated for its anticancer activity using the CCK-8 and MTT assay. Its inhibitory effect on the human non-small cell lung cancer cell line A549 was assessed following the method described in our previous study (Orabi et al., 2022; Wang et al., 2024).

### 2.10 Extraction and analysis of metabolites

*N*. ‘Blue Bird’ was selected for metabolomics as it showed superior bioactivity and PGG content among the four cultivars, making it the most informative choice for this analysis. The samples of *N.* ‘Blue Bird’ were equally divided into two aliquots, which were separately subjected to the sample preparation workflows prior to detection on the GC–MS and LC-MS/MS platforms, respectively. The methods used in this study were adapted from the previously published procedure by Alencar Filho et al. (2018) with minor modifications. A solid sample weighing 50 mg was precisely measured and placed into a 2 mL centrifuge tube, with a 6 mm grinding bead added for LC-MS/MS analysis. Metabolites were extracted by adding 400 μL of extraction solvent (water: methanol = 1:4, v/v) with 0.02 mg/mL of the internal standard L-2-chlorophenylalanine. Using a Wonbio-96c Frozen Tissue Grinder (Wanbo Biotechnology Co., Ltd., Shanghai, China), the samples were ground for 6 min at −10 °C and 50 Hz, followed by a 30-min low-temperature ultrasonic extraction at 5 °C and 40 kHz. Following a 30-min incubation at −20 °C, the samples underwent centrifugation at 13,000 rpm and 4 °C for 15 min, with the supernatant being transferred to vials for LC-MS/MS analysis. A precise 50 mg of solid sample was placed into a 2 mL grinding tube, followed by the addition of 500 μL of a methanol-water mixture (CH_3_OH: H_2_O = 4:1, *v*/v) with 0.02 mg/mL ribitol as the internal standard for GC–MS analysis. A stainless steel grinding ball and an extra 200 μL of chloroform were added to the tube, and the samples underwent frozen grinding at 50 Hz for 3 min (repeated twice). The mixtures were subjected to low-temperature ultrasonication for 30 min, followed by a 30-min incubation at −20 °C, then centrifuged at 13,000 rpm and 4 °C for 15 min. The supernatant was transferred into glass derivatization vials and evaporated to dryness using nitrogen gas. Next, 80 μL of a methoxypyridine hydrochloride solution at a concentration of 15 mg/mL was introduced to each vial. After shaking for 2 min, the oximation reaction took place in a shaking incubator set to 37 °C for 90 min. The solution was then extracted, and 80 μL of BSTFA derivatization reagent with 1% TMCS was introduced. After 2 min of vortexing, the silylation reaction was performed at 70 °C for 60 min. Finally, the derivatized samples were then left to equilibrate at room temperature for 30 min before undergoing GC–MS analysis.

Samples for LC-MS/MS analysis were divided using an ACQUITY HSS T3 column (100 mm × 2.1 mm, 1.8 μm, Waters, Milford, MA, USA) connected to a UHPLC-Q Exactive HF-X system (Thermo Fisher Scientific, Waltham, MA, USA), while GC–MS analysis was performed with a TRACE 1610 gas chromatograph connected to an Orbitrap Exploris mass spectrometer (Thermo Fisher Scientific, Waltham, MA, USA). The LC-MS and GC–MS non-target metabolomics data matrices were combined, and duplicate metabolites were eliminated. After eliminating duplicate metabolites, the data matrix was uploaded to the Majorbio cloud platform (https://cloud.majorbio.com) for analysis.

### 2.11 Examination of the weighted gene correlation network

We used weighted gene correlation network analysis (WGCNA) to explore the relationships between metabolite co-expression modules and biochemical traits. Metabolites that exhibited similar correlation and co-expression patterns were grouped into separate modules, each given a specific color, whereas those that couldn't be grouped were placed in the gray module. Eigen-metabolites (MEs) for each module, reflecting the average metabolite expression, were calculated and linked to the measured levels of bioactive compounds and important biological traits. Ultimately, a network visualization of interactions among differentially abundant metabolites (DAMs) within the red, yellow, and gray modules was performed. The analysis was carried out using the majorbio cloud platform online.

### 2.12 Statistical analysis

All measurements were performed in triplicate as technical replicates, with results shown as mean ± standard deviation (SD). SPSS 27.0 software was used for statistical analysis. Group differences were evaluated using one-way ANOVA, where statistical significance was assigned to *P* < 0.05. GraphPad Prism 8.4.2 was used for graph preparation.

## 3 Results

### 3.1 The impact of drying techniques on the color in the *N*. *Species* petals

The color of food is a major quality factor that impacts consumer acceptance. In the CIE Lab color space, The L* value measures brightness, spanning from 0 (black) to 100 (white). The a* value indicates the red-green spectrum, with positive values indicating red and negative values indicating green. The b* value measures the yellow-blue spectrum, with positive values indicating yellow and negative values indicating blue. The L*, a*, b* values were assessed to evaluate how various drying techniques impact the color parameters of water lily petals. Fig. 1E–H illustrates that the L* values for FP and FD samples exceeded those for SD, HD, and MD samples across all four cultivars. The a* color value was greater in FD and FP samples compared to those processed with other drying methods for *N*. ‘Ruby’. This could be due to FD maintaining natural lightness and redness, likely because of its lower drying temperature and lack of localized heat spots (Qiu et al., 2020). For the *N.* ‘Eldorado’ cultivar, drying increased the b* value of the petals, with HD, MD, and SD having notably higher b* values than FD and FP, indicating a more pronounced yellow color. On the other hand, the negative b* value for MD in *N*. ‘Blue Bird’, *N*. ‘Purpledo’, and *N*. ‘Ruby’ was higher (more negative) than in other drying methods, implying that MD intensifies the blue hue of the petals. This result stems from a physico-chemical synergistic effect, which may make microwave drying a promising method for producing blue hues in dried flowers.

### 3.2 The impact of drying techniques on the shrinkage of *N*. species petals

The *N.* ‘Eldorado’, ‘Blue Bird’, ‘Purpledo’ and ‘Ruby’ petals were dried using FD, SD, HD and MD methods. As shown in Fig. 1E-H, each sample exhibited a different degree of shrinkage, with FD samples shrinking the least, then MD samples. This occurred because the ice within the cell turned into water vapor and escaped in a vacuum, leaving the space once filled by the frozen water unchanged, resulting in minimal shrinkage of the FD sample, which aligns with the conclusion of Qiu et al. (2020). Shrinkage during microwave drying occurs because water inside the material rapidly vaporizes, generating high-pressure steam that pushes open the material's structure. This porous framework formed provides strong mechanical support for the material, effectively resisting the contraction forces caused by moisture loss (Joardder et al., 2017; Zhang et al., 2006; Zhou et al., 2025). The shrinkage rate of SD and HD samples was very close to each other, but higher than that of the FD and MD samples in four varieties of *N. species* petal. Heat makes the process gentle and uniform during indoor shade drying. During hot air drying, the surface evaporation rate significantly exceeds the internal moisture diffusion rate (Nwankwo et al., 2023). Both drying methods cause the material to collapse as moisture is lost.

### 3.3 The impact of drying techniques on the volatile odor in the *N*. *species* petals

The e-nose was employed to replicate the human olfactory system and can also identify the volatile scents of complex ingredients (Peris & Escuder-Gilabert, 2009). In processing e-nose data, principal component analysis (PCA) is the most frequently utilized method. Fig. 2 shows PC1 and PC2 together explained 91–96.6% of the total variance among cultivars, with PC1 being the major contributor (76.4–89.2%) to the differentiation of samples, highlighting its significant discriminatory ability between fresh petals and drying-treated petals. For four water lily varieties, FP formed distinct clusters that were clearly separated from all dried samples along PC1. The dried samples (SD, MD, HD) were grouped closely, whereas the FD samples were in a separate area, near the fresh petals for *N*. ‘Blue Bird’, *N*. ‘Purpledo’ and *N*. ‘Ruby’, suggesting that FD successfully maintained the petal's characteristic floral scent (Fig. 2A-D).

**Fig. 2:**
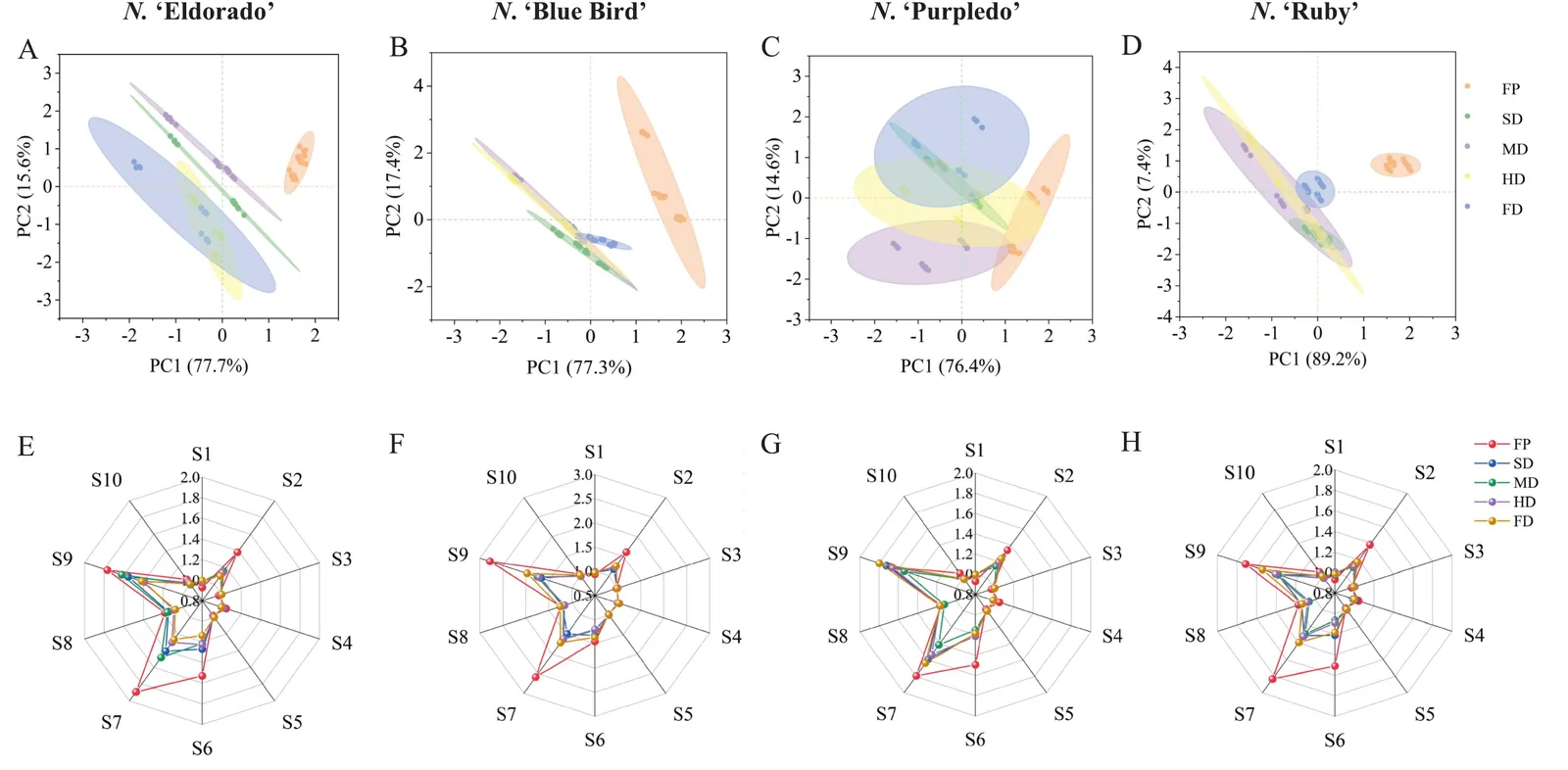
Principal component analysis (PCA) (A-D) and the radar chart (E-H) of water lily petals dried by four different drying methods. Abbreviations: FP, fresh petal; SD, indoor shade drying; MD, microwave drying; HD, hot air drying; FD, freeze drying.

A lower drying temperature effectively prevents the loss or destruction of odor, maintaining the natural aroma of food products (Qiu et al., 2020). For *N*. ‘Eldorado’, the MD samples were closely aligned with the fresh petals, indicating that the petals' scent was well preserved. In Fig. 2E-H, the radar chart for dried water lily petals is illustrated. It shows that the FP signals for S7 or S9 in all dried samples were more pronounced than others. Table 1 reveals that S7 and S9 sensors are responsive to sulfur compounds, which may be the reason for the unique fragrance of water lilies. Consistent with the results of PCA, the radar plots also reveal that FD effectively preserved the characteristic aroma profiles of *Nymphaea* ‘Blue Bird’, ‘Purpledo’, and ‘Ruby’, maintaining signal patterns that closely resembled those of fresh petals. In contrast, the aroma retention effect of FD was less pronounced in *Nymphaea* ‘Eldorado’ (Fig. 2E-H).

### 3.4 The impact of drying techniques on the total polyphenols, flavonoids, and anthocyanins content in the *N*. *species* petals

According to Ioannou et al. (2012), thermal processes can have a substantial impact on flavonoids. Fig. 3 shows the levels of polyphenols, flavonoids, and anthocyanins in water lily petals dried using various methods. FP exhibited the lowest total polyphenols, flavonoids, and anthocyanins content across all four cultivars, providing a clear baseline for the impact of drying treatments. Overall, the total polyphenol content (TPC) in dried *Nymphaea* petals showed no significant variation among the four drying techniques, except in the case of *N*. ‘Purpledo’, where HD produced a notably lower TPC than MD and FD (Fig. 3A-D). For total flavonoid content (TFC), SD produced the highest levels in all three cultivars tested, except for *N*. ‘Ruby’, where it yielded significantly lower TFC than MD (Fig. 3E-H). Regarding total anthocyanin content (TAC), SD and FD treatments led to higher TAC in three cultivars relative to MD and HD, while this trend did not apply to *N.* ‘Eldorado’ (Fig. 3I-L). *N*. ‘Eldorado’ showed the greatest total flavonoid content among the four cultivars at 2.54 ± 0.12 mg/g, although its total anthocyanin content was lower compared to the other cultivars.

**Fig. 3:**
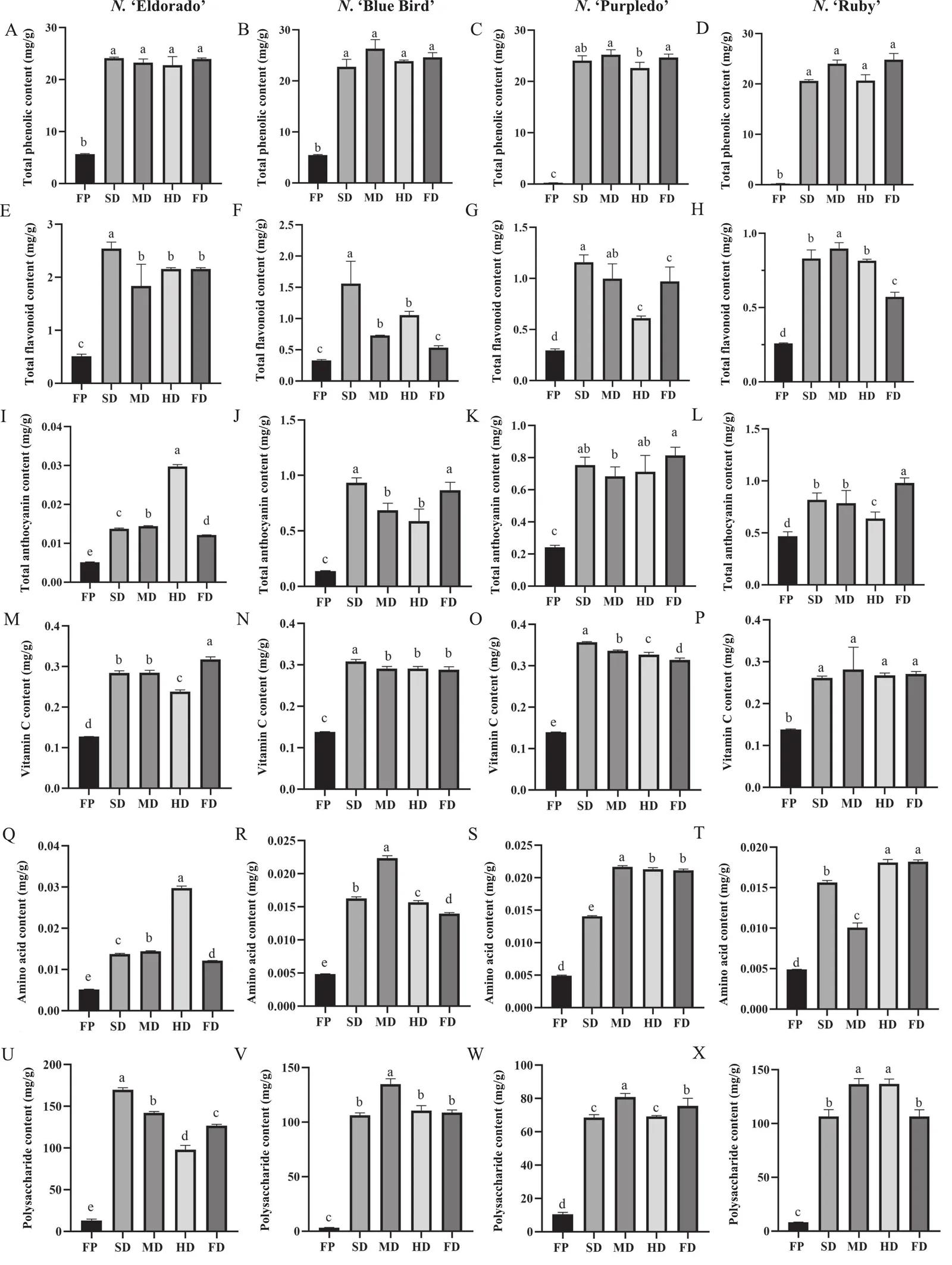
The impact of four drying techniques on the total phenolic (A-D), total flavonoids (E-H), total anthocyanins (I-L), vitamin C (M-P), amino acids (Q-T), and polysaccharides (U-X) content in petals of four *Nymphaea* cultivars. Distinct lowercase letters denote significant differences between treatments (*P* < 0.05). Values are presented as the mean ± SD from three replicates. Abbreviations: FP, fresh petal; SD, indoor shade drying; MD, microwave drying; HD, hot air drying; FD, freeze drying.

### 3.5 The impact of drying techniques on the contents of amino acid, vitamin c and polysaccharide content in the *N*. *species* petals

Vitamin C (VC) is a crucial nutrient for both humans and animals, playing a role in blood pressure regulation and the prevention of arteriosclerosis. In plants, VC serves as a substrate for enzymes that eliminate reactive oxygen species, safeguarding the plant from oxidative damage (Shi et al., 2017). The room-dried extracts of *Hibiscus sabdariffa* leaf powder contained more vitamin C compared to those that were oven-dried, crossflow-dried, microwave-dried, freeze-dried, sun-dried and infrared-dried (Kumar et al., 2015). Our study demonstrated that vitamin C content in petals was closely associated with the SD treatment. Out of the four drying techniques, SD led to a higher VC content in water lily petals, except for the *N*. ‘Eldorado’ cultivar, where SD showed significantly lower VC levels compared to FD. Among the four water lily cultivars examined, *N*. ‘Purpledo’ exhibited the highest VC content at 0.36 ± 0.0014 mg/g (Fig. 3M-P).

Amino acid (AA) serves as the precursor for protein synthesis. Microwave and oven drying methods can enhance the amino acid content in *Flos lonicerace* (Juyan et al., 2006). Notably, the hot air-dried product had higher levels of Asp, Phe, Tyr, and Ser compared to fresh mushrooms, possibly due to the release of some free amino acids during proteolysis (Yang et al., 2020). In this research, MD treatment resulted in the highest total amino acid content in *N.* ‘Blue Bird’ and *N.* ‘Purpledo’ compared to other drying methods, both reaching up to 0.022 mg. Additionally, *N.* ‘Eldorado’ and *N.* ‘Ruby’ accumulated considerable amounts of amino acids under HD treatment, attaining 0.030 mg/g and 0.018 mg/g, respectively (Fig. 3Q-T). These findings align with previous reports in the literature.

MD consistently led to the greatest accumulation of total polysaccharides among the cultivars, with the most significant increases seen in *N*. ‘Blue Bird’ (134.95 ± 4.81 mg/g), *N*. ‘Ruby’ (136.65 ± 5.19 mg/g), and *N*. ‘Purpledo’ (80.99 ± 2.05 mg/g). In *N*. ‘Eldorado’, SD produced the most polysaccharides (169.67 ± 2.56 mg/g), with MD and FD following. MD-treated *Hovenia dulcis* peduncles had higher polysaccharide yields than those treated with FD, HD, and VD (Liu et al., 2020). MD might be an effective method for drying to extract bioactive polysaccharides from loquat leaves (Fu et al., 2020). The microwave causes high vapor pressure and temperature on cell wall polymers, aiding in the efficient extraction of polysaccharides (Guo et al., 2024). Moreover, higher microwave power is associated with greater polysaccharide production (Fu et al., 2020; Liu et al., 2020).

### 3.6 The impact of drying techniques on the antioxidant activities in the *N*. *species* petals

The antioxidant capacities of four *Nymphaea* cultivars including fresh petals and treated petals with four drying were assessed using ABTS and DPPH radical scavenging assays, with results presented as IC₅₀ values (lower values denote stronger antioxidant activity) (Table S2). SD showed the lowest DPPH IC₅₀ value (3.50 ± 0.96 μg/mL), suggesting it had the most potent DPPH scavenging activity, whereas MD demonstrated the highest ABTS radical scavenging activity with an IC₅₀ of 0.74 ± 0.63 μg/mL in *N*. ‘Eldorado’. The MD extract from *N.* ‘Blue Bird’ showed the strongest antioxidant activity, with IC₅₀ values of 0.08 ± 0.04 μg/mL for DPPH and 1.68 ± 0.66 μg/mL for ABTS. In *N*. ‘Purpledo’, SD achieved the lowest DPPH IC₅₀ value, and MD had the highest ABTS scavenging activity. FP showed the greatest DPPH radical scavenging capacity, whereas FD had the most effective ABTS radical scavenging in *N*. ‘Ruby’. Overall, the results suggest that the drying techniques and *Nymphaea* varieties have a notable impact on antioxidant activity, with both MD and SD boosting DPPH radical scavenging effects, and MD improving ABTS radical scavenging effects. Like the patterns seen in DPPH and ABTS radical scavenging tests, MD typically enhanced the ferric reducing antioxidant power (FRAP) in *N*. ‘Blue Bird’ and ‘Ruby’ (Fig. S1).

### 3.7 The impact of drying techniques on the other biological properties in the *N*. *species* petals

Among the drying treatments, FD exhibited the most effective tyrosinase inhibitory activity in *N.* ‘Purpledo’, while MD showed the most potent activity in the other three *Nymphaea* varieties. Furthermore, the tyrosinase inhibitory activity of *N.* ‘Purpledo’ was higher than that of the other *Nymphaea* varieties. SD produced the greatest α-amylase inhibitory activity in *N.* ‘Eldorado’ and *N.* ‘Ruby’, whereas FD led to the highest activity in *N.* ‘Blue Bird’ and *N.* ‘Purpledo’ among the dried samples. Among all four varieties, the freeze-dried *N.* ‘Blue Bird’ sample achieved an α-amylase inhibitory rate of 82.66 ± 0.35%, which was higher than that of the positive control group at the same concentration. The pancreatic lipase inhibitory rates differed with various drying methods, and *N.* ‘Blue Bird’ showed the highest pancreatic lipase inhibitory rate under SD (Fig. 4A-L). For antimicrobial activity, all extracts inhibited *Escherichia coli*, *Staphylococcus aureus*, and *Pseudomonas aeruginosa* as evidenced by inhibition zones, with the most pronounced effects observed against *P. aeruginosa*. Drying method impacts on antimicrobial activity were cultivar-specific, with MD extracts displaying larger inhibition zone diameters relative to other drying methods against *P. aeruginosa* in some varieties (Fig. 4M–P).

**Fig. 4:**
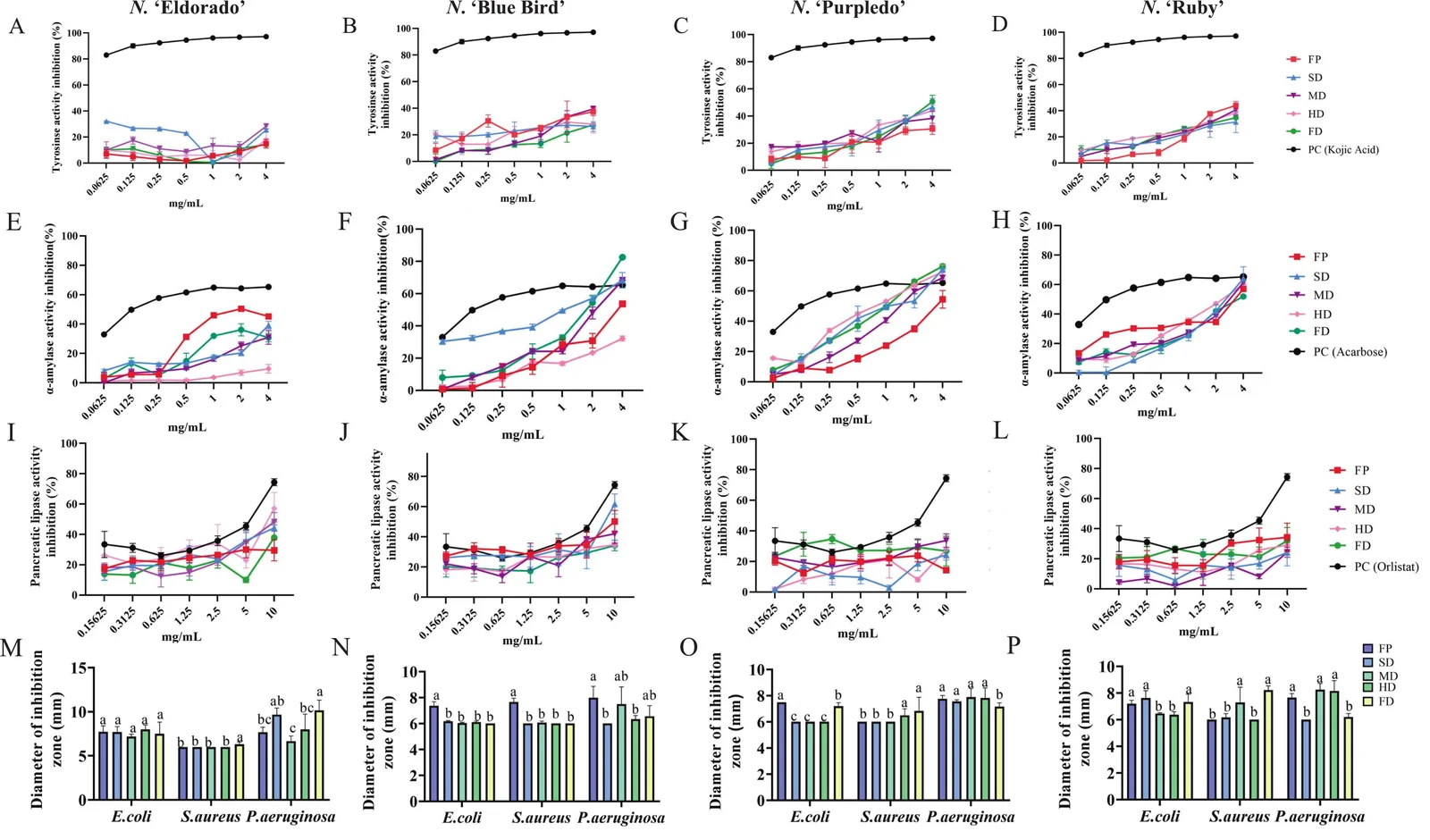
The bioactivities of four *N*. cultivars were evaluated across treatments. All extracts exhibited concentration-dependent inhibitory effects on tyrosinase (A-D), α-amylase (*E*-H), pancreatic lipase (I-L) with positive controls (PC: kojic acid, acarbose, orlistat, respectively), and antimicrobial activity (M–P). Abbreviations: FP, fresh petal; SD, indoor shade drying; MD, microwave drying; HD, hot air drying; FD, freeze drying. Data are presented as mean ± SD from three replicates. Distinct lowercase letters denote significant differences between treatments (*P* < 0.05).

### 3.8 The impact of drying techniques on major phenolic compound contents in the *N*. *species* petals

Chromatograms of phenolic extracts from petals, together with those of the standard substances, are presented in Fig. S2. Phenolic extracts were subjected to UPLC-DAD analysis with the wavelength at 260 nm. According to commercial standards, peaks S1, S2, S3, S4 were identified as gallic acid, methyl gallate, geraniin, 1,2,3,4,6-*O*-pentagalloylglucose content, respectively (Fig. S2). MD yielded the highest gallic acid content in *N*. ‘Eldorado’ (Fig. 5A), *N*. ‘Blue Bird’ (Fig. 5B) and *N*. ‘Ruby’ (Fig. 5D), and *N.* ‘Purpledo’ (Fig. 5C) displayed the highest contents under FD. Specifically, under SD treatment, *N*. ‘Eldorado’ (Fig. 5E) and *N*. ‘Purpledo’ (Fig. 5G) had the highest overall methyl gallate contents, reaching 1.69 ± 0.03 mg/g and 4.89 ± 0.02 mg/g, respectively. In contrast, *N*. ‘Blue Bird’ (Fig. 5F) and *N*. ‘Ruby’ (Fig. 5H) achieved peak methyl gallate levels under FD treatment, with values of 2.81 ± 0.11 mg/g and 2.59 ± 0.43 mg/g. Geraniin showed the more dramatic accumulation in SD samples (Figs. 5I–L). For 1,2,3,4,6-*O*-pentagalloylglucose (PGG) (Fig. 5M–P), its content varied significantly depending on the *Nymphaea* cultivar and drying method employed. Specifically, *N*. ‘Blue Bird’ (Fig. 5N) and *N.* ‘Purpledo’ (Fig. 5O) showed the highest PGG contents under SD treatment, whereas *N.* ‘Ruby’ (Fig. 5P) achieved peak PGG levels under FD treatment. In contrast, *N*. ‘Eldorado’ (Fig. 5M) accumulated relatively higher PGG content under HD treatment. In summary, SD treatment generally emerged as the most effective method for preserving or enhancing the contents of methyl gallate, geraniin, and PGG across most *Nymphaea* cultivars, while MD treatment was found to increase gallic acid contents.

**Fig. 5:**
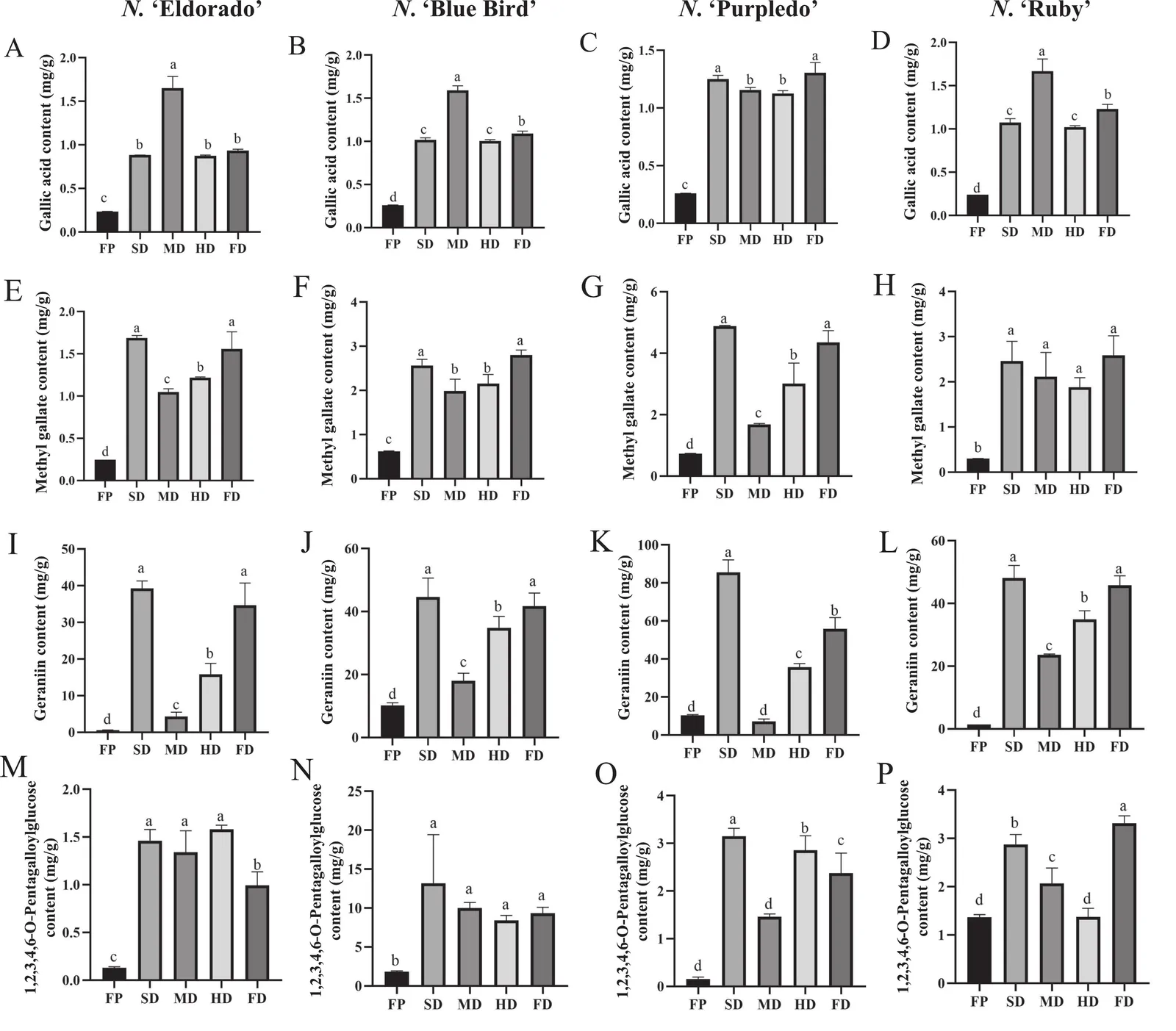
Concentrations of individual phenolic components in fresh and dried petals of *Nymphaea* cultivars. A-B, Gallic acid content; E-H, Methyl gallate content; I-L, Geraniin content; M-P, 1,2,3,4,6-*O*-Pentagalloylglucose content. Data are presented as mean ± SD from three replicates. Distinct lowercase letters denote significant differences between treatments (*P* < 0.05).

### 3.9 Biological activities of major phenolic compounds in the *N*. *species* petals

All four test compounds showed dose-dependent tyrosinase inhibition. Among them, PGG exhibited the most potent activity, followed by methyl gallate, while geraniin and gallic acid displayed relatively weaker effects (Fig. S3A-D). PGG exerted clear dose-dependent inhibition of α-amylase, with the former approaching the activity of the positive control acarbose. Geraniin showed moderate activity, whereas methyl gallate and gallic acid exhibited slight enzyme activation (Fig. S3E-H). Notably, gallic acid exhibited higher pancreatic lipase inhibitory activity than the other three active components (Fig. S3I-L). PGG exhibits the highest pancreatic lipase inhibitory activity at 0.03125 mg/ml, and the average inhibition rate reaches 42.32% (Fig. S3L). PGG inhibited A549 cell growth at 0.5–1 mg/mL, suggesting it may have an anti-cancer effect (Fig. S3M-N). Methyl gallate, gallic acid, geraniin, and PGG all showed strong antioxidant activity which DPPH radical scavenging rates exceeded 90%, ABTS radical scavenging rates approached 100%, and ferric reducing antioxidant power (FRAP) values increased with concentration, reaching approximately 1.0–1.2 mmol/L (Fig. S4).

### 3.10 Correlation analysis of bioactive compounds and biological activities

Correlation analysis further delineated the interplay between biochemical traits and functional phenotypes (Fig. 6A). TPC, TFC, AA, VC, polysaccharide, gallic acid, and PGG all exhibited significant positive correlations with DPPH radical scavenging activity. α-Amylase inhibition was significantly and positively associated with TAC, FRAP, methyl gallate, gallic acid, and PGG. AA showed positive correlations with VC, polysaccharide, TPC, and TFC. VC also correlated positively with AA, TPC, and TAC, whereas polysaccharide showed positive links to TPC and AA. These collective correlations point to potential synergistic interactions between AA, VC, polysaccharides, and phenolic compounds in boosting antioxidant activity.

**Fig. 6:**
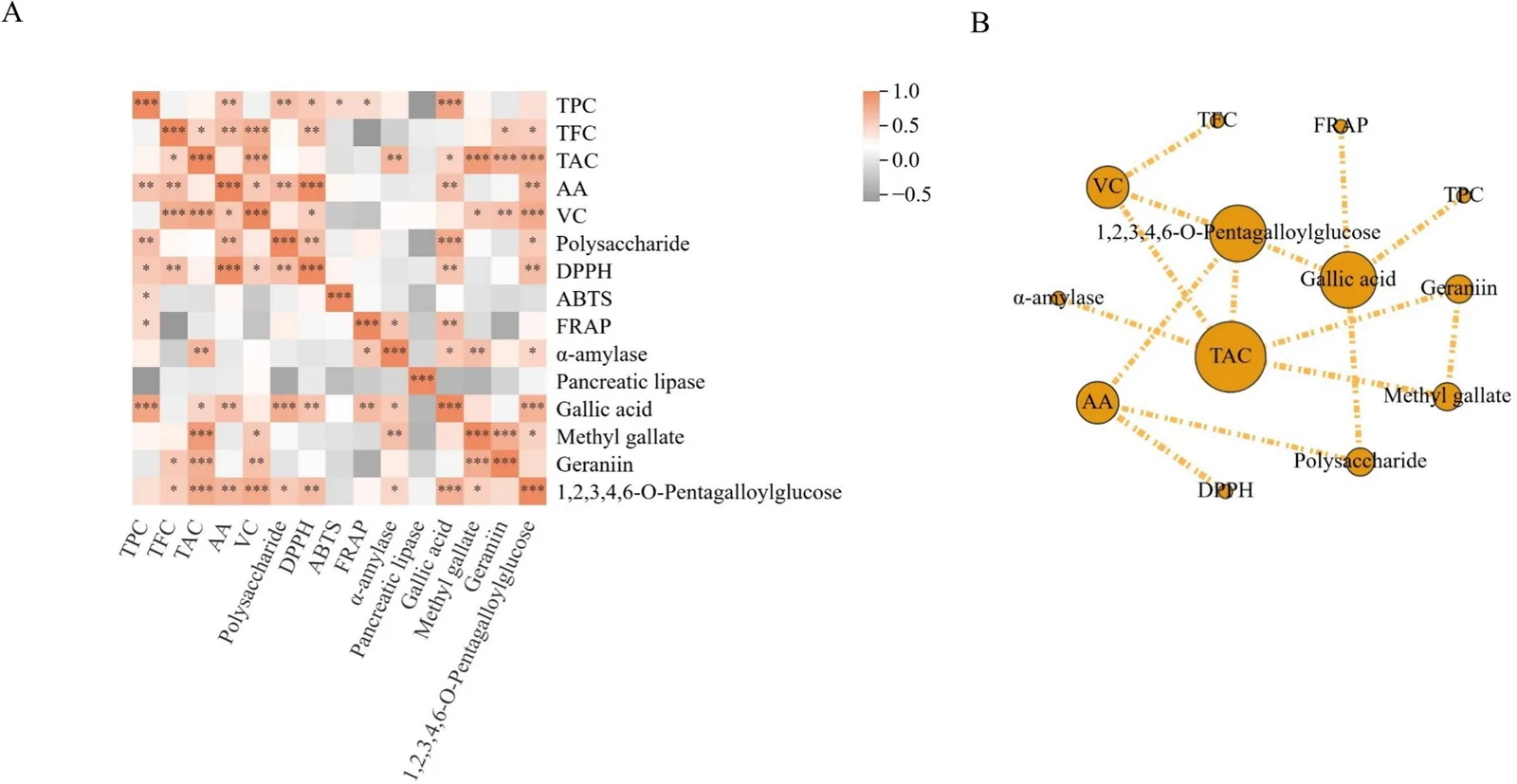
Correlation analysis and network visualization of bioactive compounds and biological activities. (A) Pearson correlation heatmap illustrating the relationships between target phenolic compounds and functional traits, with asterisks indicating statistical significance (*P < 0.05, **P < 0.01, ***P < 0.001). (B) Network visualization of compound-trait associations, where nodes represent compounds or biological traits, and edges denote significant correlations, orange-yellow edges denote positive correlations. (For interpretation of the references to color in this figure legend, the reader is referred to the web version of this article.)

Positive correlation network analysis showed that TAC, PGG, and gallic acid serve as core hub nodes (Fig. 6B). These hubs connect total TPC, VC, AA, polysaccharide, α-amylase activity, and DPPH radical scavenging activity. Node size corresponds to the connectivity of each trait or compound within the network. These results demonstrate that anthocyanins, gallic acid, PGG, other phenolic compounds, and non-phenolic bioactive substances act synergistically to boost antioxidant activity and modulate enzyme inhibitory activity.

### 3.11 Impact of various drying techniques on the metabolome

The metabolite profiles of *N*. ‘Blue Bird’ were analyzed using non-targeted metabolomics with full-MS/MS analysis, identifying a total of 3192 metabolites (Table S3). Of these, 2765 metabolites were detected and annotated via LC-MS analysis, while 427 metabolites were identified through GC–MS analysis. Flavonoids were the most abundant metabolite class across all samples, with counts increasing from 218 in fresh petals to 228 in all dried samples. Carbohydrates and derivatives, lipids, amino acids and derivatives, phenolic acids and derivatives, and terpenoids generally showed higher metabolite counts in dried samples compared to fresh petals (Fig. S5A). The correlation heatmap (Fig. S5B) revealed tight clustering of samples by preparation method, with strong within-group correlations. FP samples formed a distinct cluster, while SD and HD samples also formed a single cluster. These findings confirm that sample preparation is a key driver of metabolite profile variation, clearly separating fresh from dried samples and distinguishing between drying techniques.

### 3.12 Differentially accumulated metabolites (DAMs) between drying and FP groups

Compared to FP, 1181, 1202, 1236, and 1228 DAMs were found in the MD_vs_FP, HD_vs_FP, SD_vs_FP, and FD_vs_FP comparisons (Fig. 7A), respectively, with 221/960, 181/1021, 198/1038, and 208/1020 metabolites being down-/up-regulated (Fig. S6). Out of these, 788 DAMs were found in all comparisons, whereas 127, 33, 58, and 67 DAMs were specific to MD_vs_FP, HD_vs_FP, SD_vs_FP, and FD_vs_FP, respectively (Fig. 7A). Flavonoids exhibited the highest number of differentially accumulated metabolites (DAMs) across all drying method comparisons, followed by lipids, carbohydrates and derivatives, phenolic acids and derivatives, and amino acids and derivatives (Fig. 7B).

**Fig. 7:**
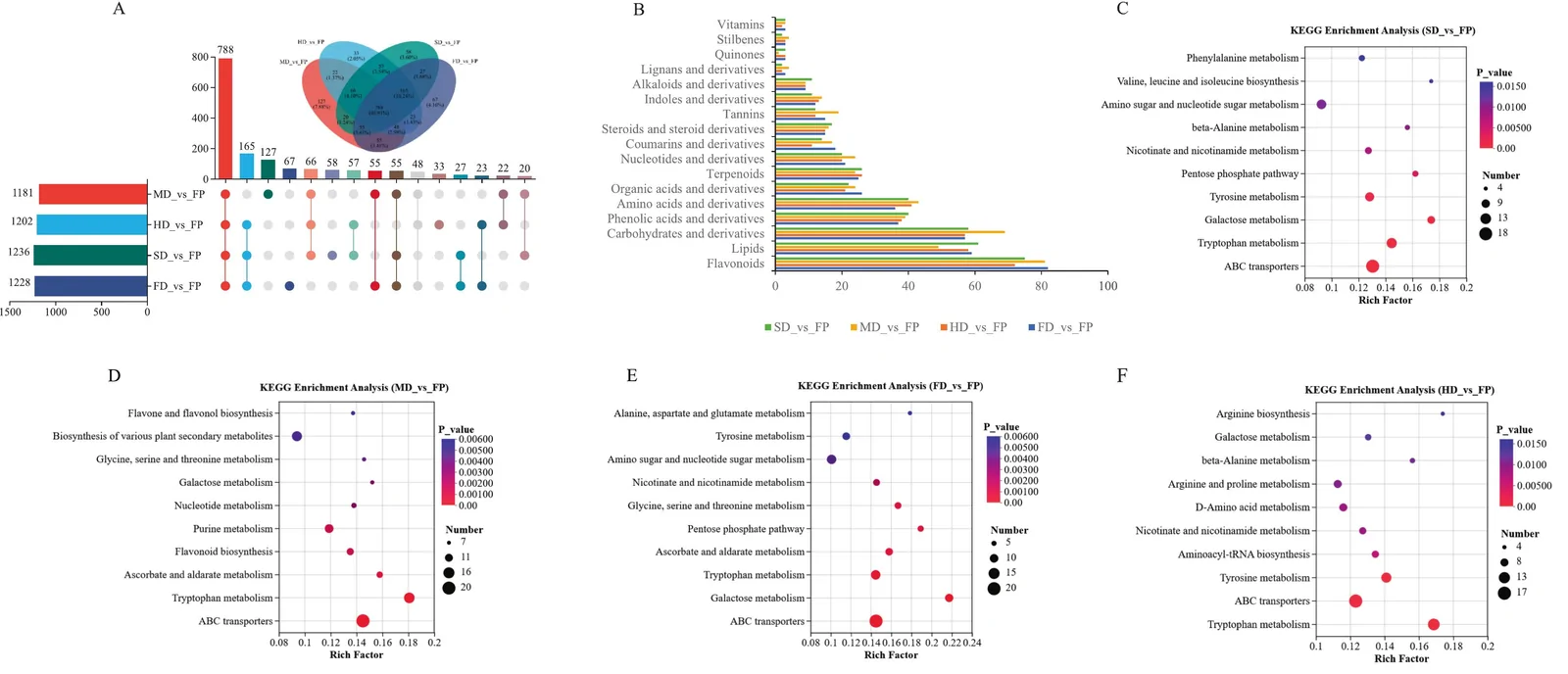
(A) An UpSet plot showing both shared and unique DAMs between the drying and FP groups. (B) The categorization and enumeration of DAMs between the drying and FP groups. (C-F) KEGG enrichment analysis comparing drying and CK groups, including SD_vs_FP (C), MD_vs_FP (D), FD_vs_FP (E) and HD_vs_FP (F). The color of the dot indicates the *P*-value, while the size of the dot indicates the number of DAMs.

Analyzing the pathways of DAMs indicated distinct metabolic reprogramming patterns for each drying method. In SD_vs_FP, DAMs were primarily enriched in ABC transporters, tryptophan metabolism, galactose metabolism, and tyrosine metabolism (Fig. 7C). In MD_vs_FP, ABC transporters and tryptophan metabolism, ascorbate and aldarate metabolism, and flavonoid biosynthesis showed enrichment (Fig. 7D). FD_vs_FP comparisons highlighted ABC transporters, galactose metabolism, tryptophan metabolism, and ascorbate and aldarate metabolism (Fig. 7E). For HD_vs_FP, ABC transporters, tryptophan metabolism, and tyrosine metabolism were predominantly enriched (Fig. 7F).

### 3.13 Impact of various drying techniques on metabolite co-expression

We created a weighted correlation network with a significance threshold of *P* < 0.05 to combine physiological, biochemical, and metabolomic datasets. After dynamic tree merging, ten co-expression modules were discovered (Fig. 8A). The MEred showed a positive correlation with tyrosinase inhibitory activity. MEturquoise showed a positive correlation with FRAP and α-amylase inhibition, whereas MEbrown exhibited a negative correlation with these activities (Fig. 8B). Further enrichment analysis of DAMs in key modules highlighted functional specialization. MEred was primarily associated with fatty acid degradation, linoleic acid metabolism, phenylalanine, tyrosine and tryptophan biosynthesis (Fig. 8C). MEturquoise was enriched in ABC transporters, tryptophan metabolism, nicotinate and nicotinamide metabolism, and tyrosine metabolism (Fig. 8D).

**Fig. 8:**
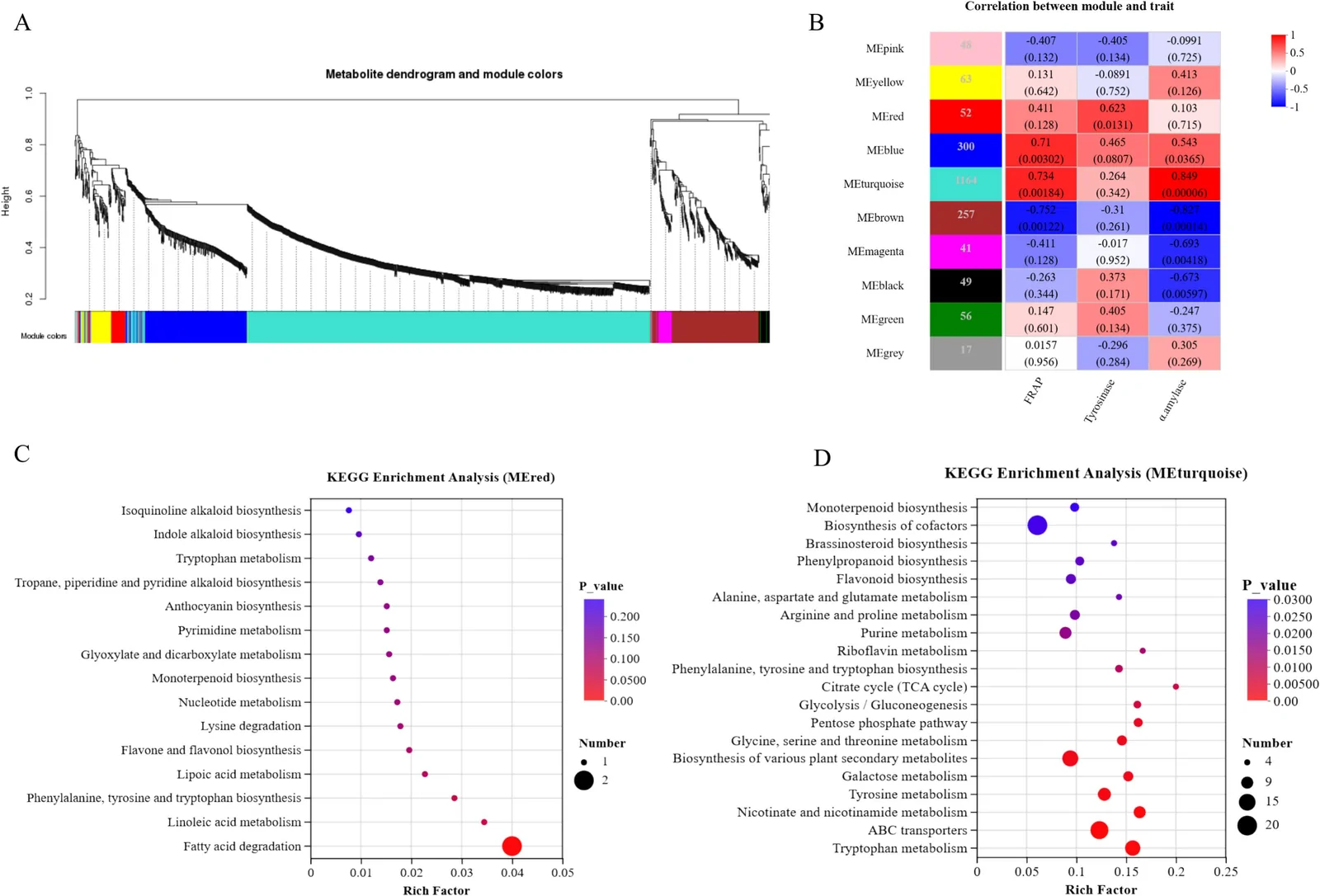
Analysis of DAMs using weighted gene co-expression network analysis (WGCNA). (A) The cluster dendrogram identifies co-expression modules, with each represented by a unique color. Metabolite clusters shown in various colors indicate unique co-expression modules, while DAMs not fitting into any co-expressed module are placed in the gray module. (B) The heatmap displays the correlation between DAMs within modules and biological activity. (C-D) The analysis of metabolic pathway enrichment in Mered (C) and Meturquoise (D). The color of the dots indicates the *p*-value, while their size shows the number of DAMs.

## 4 Discussion

Water lilies are known for their rich content of bioactive compounds with various physiological functions, making them valuable raw materials for developing functional foods (Raja et al., 2010). To enhance their health benefits, prolong shelf life, and meet increasing consumer demand, we systematically assessed the impact of various drying methods. All four drying methods tested are suitable for commercial dried flower production, but they caused significant differences in flower morphology and aroma. MD, SD, and HD led to darker flower color, more severe shrinkage, and reduced aroma, while FD effectively maintained the flowers' original color, morphology, and aroma. These results align with earlier studies indicating that vacuum freeze-drying is more effective at maintaining the green color and natural taste of fresh fruits (Li et al., 2026). Notably, the superior volatile profile of fresh petals over dried samples at the same raw material weight strongly suggests that the drying protocols caused substantial degradation or loss of aroma compounds, outweighing any concentration effect arising from dehydration.

Compared with fresh petals, drying treatments led to increased levels of TPC, TFC, TAC, AA, vitamin C, and polysaccharides in water lily petals, along with enhanced antioxidant capacity (DPPH, ABTS, FRAP). Accordingly, these differences may be partially explained by a concentration effect caused by the removal of water during the drying process. In most cultivars, MD resulted in higher contents of TPC, AA, and polysaccharides, while SD improved VC and TFC levels compared to other drying methods. In terms of antioxidant activity, SD and MD both boosted DPPH free radical scavenging activity, whereas MD specifically increased ABTS radical scavenging activity and FRAP values. Additionally, MD showed higher inhibitory rates against tyrosinase activity and *Pseudomonas aeruginosa* across the tested *Nymphaea* varieties. These bioactive components are closely linked to the biological activities of water lily petals. That said, some statistically significant differences were small in magnitude and may not be biologically meaningful.

Our findings are supported by previous work. Freeze-drying best preserved flavonoid content and antioxidant capacity in *Salix songarica* flowers (Dai et al., 2026). For microwave drying, Hu et al. (2023) noted that it provides the lowest operational costs and excellent preservation of bioactive components and the most diverse and abundant volatile compounds. In *Bletilla striata* flowers, natural drying required a long duration and caused severe browning, while microwave drying achieved the shortest drying time (Lu et al., 2021). Moreover, microwave vacuum drying (MVD) used as a post-freeze-drying (FD-MVD) treatment significantly increased the total phenol and flavonoid contents in *Citrus changshanensis* extracts, showing the greatest antioxidant activity (Li et al., 2020). Febrinda et al. (2023) observed that various drying techniques greatly affected *Eleutherine bulbosa* capacity to inhibit *Staphylococcus aureus*, and Lu et al. (2024) found that polyphenols extracted from vacuum-dried samples showed the strongest anti-tyrosinase activity. Taken together, our findings demonstrate that MD is a suitable drying method for water lilies, particularly in terms of retaining bioactive components and strong biological activities. Metabolomic analysis revealed flavonoids were the most responsive DAMs when comparing all dried samples to fresh petals, highlighting the vulnerability of phenylpropanoid metabolism to drying. The significant enrichment of flavonoid biosynthesis in the MD_vs_FP comparison aligns with this shift, and may account for the superior bioactivity of microwave-dried petals.

The water lilies contained methyl gallate, gallic acid, geraniin, and PGG. It is worth noting that PGG exhibited strong antioxidant properties, inhibitory effects against tyrosinase, α-amylase, and pancreatic lipase and anti-tumor activities. Anthocyanins, gallic acid, and PGG played crucial roles in the physiological characteristics and functional properties of water lilies. Anthocyanins from *Dioscorea alata* improved muscle antioxidant capacity by boosting glutathione peroxidase and catalase activities and decreasing lipid peroxidation (Yang et al., 2026). Additionally, The phytochemical gallic acid can mitigate nonalcoholic fatty liver disease by regulating both lipid metabolism and mitochondrial function (Zhang et al., 2023). Gallic acid improved symptoms of Parkinson's disease (Sheikhpour et al., 2023). PGG has gained interest due to its therapeutic potential, demonstrating anti-carcinogenic, antimicrobial, antidiabetic, anti-inflammatory, and antioxidant properties (Torres-León et al., 2017). PGG represents a promising bioactive marker in *Nymphaea* petals, with its functional significance deserving further validation.

The *N.* ‘Eldorado’ water lily is a well-liked variety in Yunnan for making herbal tea, offering a delightful flavor and high nutritional content due to its abundance of flavonoids, amino acids, and polysaccharides*. N.* ‘Blue Bird’ shows strong biological activities, with remarkable antioxidant, α-amylase inhibitory and pancreatic lipase inhibitory effects, along with a high content of the bioactive compound PGG. *N.* ‘Blue Bird’ thus serves as a promising health food candidate with antidiabetic and anti-obesity potential.

Non-targeted metabolomic profiling has further clarified its metabolic reprogramming of *N.* ‘Blue Bird’ during drying, as well as the associated underlying pathway-level responses. Variations in flower appearance and the buildup of bioactive compounds were linked to both the quantity and specificity of DAMs in SD_vs_FP, MD_vs_FP and HD_vs_FP (Li et al., 2026). The differentially accumulated metabolites in fresh petals and petals subjected to different drying treatments were mainly enriched in the ABC transporter and tryptophan metabolism pathways. Pathways associated with ABC transporters and tryptophan metabolism also showed enrichment in FRAP and α-amylase inhibitory activity, while tyrosinase inhibitory activity was associated with fatty acid degradation. Tryptophan metabolism aids in abiotic stress tolerance, delays aging, and extends shelf life via its downstream metabolites (Arabia et al., 2025; Ayyanath et al., 2024). ABC transporters are among the largest and oldest protein superfamilies found in all living organisms. These transporters use ATP hydrolysis to power the transport of molecules across the cell membrane (Chen et al., 2025). Different drying methods exert differential effects on cell membrane structure and membrane protein activity, thereby regulating the function of ABC transporters. This helps reduce the loss of bioactive components during drying and maintain the flavor, nutritional value and metabolomic characteristics of dried water lilies. Fatty acids influence the breakdown of tyrosinase, an essential enzyme linked to melanin production in melanocytes (Ando et al., 1999; Ando et al., 2004). Nevertheless, it is necessary to directly validate the functionality of these pathways.

Several limitations of this study should be noted. Only technical replicates were performed, and the absence of biological replicates limits conclusions about natural variation among individuals. Additionally, as an in vitro study, the in vivo bioactivity of the compounds was not examined. Furthermore, the mechanisms underlying the compositional changes during drying remain unexplored. These factors should be kept in mind when interpreting the results and extending them to other contexts.

## 5 Conclusions

Various drying techniques were crucial in affecting water lily quality. Comprehensive assessment of appearance and physicochemical properties showed that petals treated with FD maintain visual quality and aroma stability best, making them ideal for commercial sale and direct use. Conversely, petals treated with MD demonstrated clear benefits for enhancing and extracting particular bioactive components and biological activities of the *N*. varieties. Among the identified compounds, anthocyanins, gallic acid, and 1,2,3,4,6-*O*-pentagalloylglucose emerged as key bioactive markers for functional food use. Collectively, these findings support the selection of appropriate drying methods to extend shelf life, maintain quality, and enhance the value of *Nymphaea* flowers or diverse applications.

## CRediT authorship contribution statement

**Xuhong Zhou:** Writing – review & editing, Writing – original draft, Methodology, Funding acquisition, Conceptualization. **Jiangli Peng:** Software, Investigation, Data curation. **Qianqian Li:** Resources, Investigation. **Fujun Zheng:** Resources, Investigation. **Min Tian:** Writing – review & editing, Supervision, Conceptualization. **Suping Qu:** Supervision, Project administration, Funding acquisition.

## Declaration of competing interest

The authors declare that they have no known competing financial interests or personal relationships that could have appeared to influence the work reported in this paper.

## Data Availability

No data was used for the research described in the article.
