# Supplementary material for: An integrative evaluation of drying methods in relation to bioactive properties and metabolomic profiles of four Nymphaea cultivar petals
Source: Food Chem X. 2026 Sep 19;39:104468. doi: 10.1016/j.fochx.2026.104468 (PMC13632170; doi:10.1016/j.fochx.2026.104468)
Supplement: Supplementary file 1 [file mmc1.docx]

Fig. S1 The ferric reducing antioxidant power (FRAP) activity of extracts from fresh and dried products.

Fig. S2 HPLC chromatograms of reference substance (A) and sample (B). 1: Gallic acid; 2: Methyl gallate; 3: Geraniin; 4: 1,2,3,4,6-*O*-pentagalloylglucose.

Fig. S3 Inhibitory activities of gallic acid, methyl gallate, geraniin, and PGG against key enzymes. (A–D) Tyrosinase inhibitory activity (positive control: kojic acid); (E–H) α-amylase inhibitory activity (positive control: acarbose); (I–L) Pancreatic lipase inhibitory activity (positive control: orlistat). Effects of PGG on the viability of A549 by CCK8 (M) and MTT (N) assay . Values are presented as the mean ± SD from three replicates. ****p < 0.0001 vs. control.

Fig. S4 The antioxidant activities of gallic acid, methyl gallate, geraniin, and 1,2,3,4,6-*O*-pentagalloylglucose were assessed via DPPH (A-D) and ABTS (E-H) free radical scavenging and FRAP (I-L).

Fig. S5 Metabolomic profiling of water lily. (A) Classification and count of metabolites; (B) Correlation of metabolite accumulation between different samples.

Fig. S6 Volcano plots of differentially accumulated metabolites (DAMs) in water lily comparing different drying methods.
